# Antiretroviral therapy at a district hospital in Ethiopia prevents death and tuberculosis in a cohort of HIV patients

**DOI:** 10.1186/1742-6405-3-10

**Published:** 2006-04-07

**Authors:** Degu Jerene, Are Næss, Bernt Lindtjørn

**Affiliations:** 1 Arba Minch Hospital, Ethiopia; 2 Centre for International Health, University of Bergen, Norway; 3 Institute of Medicine, University of Bergen, Norway

## Abstract

**Background:**

Although highly active antiretroviral therapy (HAART) reduces mortality in the developed world, it remains undocumented in resource-poor settings. We assessed the effect of HAART on patient mortality and tuberculosis incidence rate under routine clinical care conditions in Ethiopia. The objective of this study was to assess the effect of HAART on patient mortality and tuberculosis incidence rate under routine clinical care conditions in a resource-limited setting in south Ethiopia. Starting in January 2003, we followed all consecutive adult HIV infected patients who visited the HIV clinic. Since August 2003, we treated patients with HAART. Only basic laboratory services were available.

**Results:**

We followed 185 patients in the pre-HAART cohort and 180 patients in the HAART cohort. The mortality rate was 15.4 per 100 person-years of observation (PYO) in the HAART group and tuberculosis incidence rate was 3.7 per 100 PYO. In the pre-HAART group, the mortality rate was 58.1 per 100 PYO and the tuberculosis incidence rate was 11.1 per 100 PYO. HAART resulted in a 65% decline in mortality (adjusted hazard ratio [95%CI] = 0.35 [0.19–0.63]; P < 0.001). Tuberculosis incidence rate was lower in the HAART group (adjusted hazard ratio [95%CI] = 0.11 [0.03–0.48]; P < 0.01). Most of the deaths occurred during the first three months of treatment.

**Conclusion:**

HAART improved survival and decreased tuberculosis incidence to a level similar to that achieved in the developed countries during the early years of HAART. However, both the mortality and the tuberculosis incidence rate were much higher in terms of absolute figures in this resource-limited setting. Attention should be paid to the early weeks of treatment when mortality is high. The high tuberculosis incidence rate, when coupled with the improved survival, may lead to increased tuberculosis transmission. This highlights the need for strengthening tuberculosis prevention efforts with the scale-up of treatment programmes

## Background

The survival benefit of highly active antiretroviral therapy (HAART) in HIV infection and its impact on the incidence of opportunistic infections have been well studied in the developed world [1-3]. In resource-poor settings, where such treatment was started only recently, limited data exist both on treatment results and on how to carry out such interventions [4-6]. As a result, the existing treatment guidelines and recommendations are based on data from the developed world [7].

As HAART is effective in treating HIV infected patients, conducting randomized, placebo-controlled trials is unethical. Thus, the next-best method of measuring the effectiveness of HAART on disease progression in new settings is cohort studies. In this study, we present data from two cohorts of treatment-naïve patients in south Ethiopia. The objective of this study was to assess the effect of HAART on patient mortality and tuberculosis incidence rate under routine clinical care conditions in a resource-limited setting in south Ethiopia.

## Results

### Baseline characteristics

We enrolled and followed 185 patients (90 men and 95 women) in the pre-HAART cohort and 180 (102 men and 78 women) in the HAART cohort. 85 patients contributed person-time for both cohorts. At baseline, the two groups were similar for age, sex, BMI or TLC categories. However, patients receiving HAART had more advanced disease. The follow-up period was longer in the HAART group [50 weeks (IQR, 21–68) vs. 19 weeks (IQR, 10–34). Table 1 compares the baseline characteristics of the two groups.

**Table 1:** Baseline characteristics of the study participants, Arba Minch Hospital, 2006

	Observation period	
Characteristic	Pre-HAART	HAART
Mean age in years (SD)	33 (8.9)	34 (9.0)
Gender; count (%)		
Male	90 (49)	102 (57)
Female	95 (51)	78 (43)
WHO stage; count (%)		
II	28 (15)	15 (8)
III	132 (71)	120 (67)
IV	25 (14)	45 (25)
Median TLC/ml (range)	1200 (200–380)	1140 (200–4800)
Mean Hgb (SD)	8.6 (2.2)	9.2 (1.9)
Oral thrush; count (%)	67 (36)	50 (28)

TLC=Total lymphocyte count; Hgb=Haemoglobin in gram per decilitre; SD=Standard deviation

### The HAART cohort

#### Patient follow-up

At the end of the study, 136 patients (76%) were under regular follow-up, 25 (13.9%) died, 9 (5%) stopped treatment, 5 (2.8%) were lost to follow-up and 5 patients (2.8%) were transferred to other health institutions. 13 out of the 25 deaths (52%) occurred at home, 11(44%) at the hospital, and in one patient place of death was not recorded. The reasons for stopping treatment were: drug related side effects (3 patients), lack of public transport to the hospital (1 patient), afraid of swallowing tablets in front of her husband (1 patient), two patients preferred taking traditional medicine ("holy water") instead of drugs, and no reason was given for two patients.

#### Antiretroviral drugs prescribed

93 patients (52%) used the first line treatment drugs (d4T/3TC/NVP) and 48 patients (27%) received d4T/3TC/EFV. Twenty-five patients (14%) got ZDV/3TC/NVP while the remaining 14 patients (8%) received ZDV/3TC/EFV. 10 patients (6%) had previous exposure to antiretroviral drugs. Of these, 8 had received triple drugs and two dual therapy. Their inclusion in the analysis did not affect the results. Twenty-six patients were on concomitant antituberculosis treatment, including 8 in their intensive phase of treatment.

#### Mortality rate

25 patients (14%) died during the follow-up. The mortality rate was 15.4 per 100 PYO (25 deaths per 162.2 PYO). After starting treatment, the median time to death was 9 weeks (IQR, 4–25). None of the patients with stage II disease died. Over half of the deaths (13/25, 52%) occurred in patients with stage IV disease.

#### Tuberculosis incidence rate

Six patients (5 men and 1 woman) developed tuberculosis during the treatment period. The median time to the diagnosis of tuberculosis was 32 weeks (IQR, 14–56). Two of the patients had smear positive pulmonary tuberculosis (PTB+), two patients had smear negative pulmonary tuberculosis (PTB-) and two patients had extra pulmonary tuberculosis (EPTB). The incidence rate of tuberculosis was 3.7 per 100 PYO (6 cases per 162.2 PYO).

#### Drug side effects

We observed 124 episodes of drug related side effects. Peripheral neuropathy was the most common side effect (48 of 124 patients, 38.7%) followed by skin rash (41/124, 33.1%) and anaemia (11/124, 8.9%). Two men taking an EFV containing regimen developed breast enlargement. One patient, taking a d4T-containing regimen, died of pancreatitis (diagnosed clinically). Anaemia was the earliest side effect recognized, occurring at a median time of 4.4 weeks (IQR, 4.4–16.6) followed by skin rash (median, 4.6 weeks; IQR, 3.6–13.0). Two patients with anaemia needed blood transfusion. Table 2 summarizes the side effects.

**Table 2:** ART associated side effects, Arba Minch Hospital, 2006

Side effect§	Frequency	Per cent	Time to first episode (weeks)	
			Median	IQR
Peripheral neuropathy	48	38.7	13.1	5.9–23.6
Skin rash	41	33.1	4.6	3.6–13.0
Anaemia	11	8.9	4.4	4.0–16.6
CNS manifestations	10	8.1	5.7	3.1–12.1
Hepatotoxicity	7	5.6	9.8	4.3–12.6
Lactic acidosis	2	1.6	-	-
Abnormal fat accumulation	2	1.6	-	-
				
Gynecomastia	2	1.6	-	-
Pancreatitis	1	0.8	-	-

§ Gastrointestinal symptoms such as diarrhea, vomiting, nausea, etc were not included because it was difficult to distinguish them from disease symptoms

### The pre-HAART follow-up

At the end of the pre-HAART period, 10 patients (5.4%) were lost to follow-up, 8 (4.3%) were transferred to another health institution, 47 (25.4%) died and 120 (64.9%) patients were under regular follow-up. 38 out of the 47 deaths (81%) occurred at home, 8 (17%) at the hospital and for one patient place of death was not recorded. The pre-HAART mortality rate was 58.1 per 100 PYO (47 deaths per 80.9 PYO) and the tuberculosis incidence rate was 11.1 per 100 PYO (9 cases per 80.9 PYO).

### The pre-HAART vs. HAART cohorts: comparison of mortality and tuberculosis incidence

**Figure 1:**
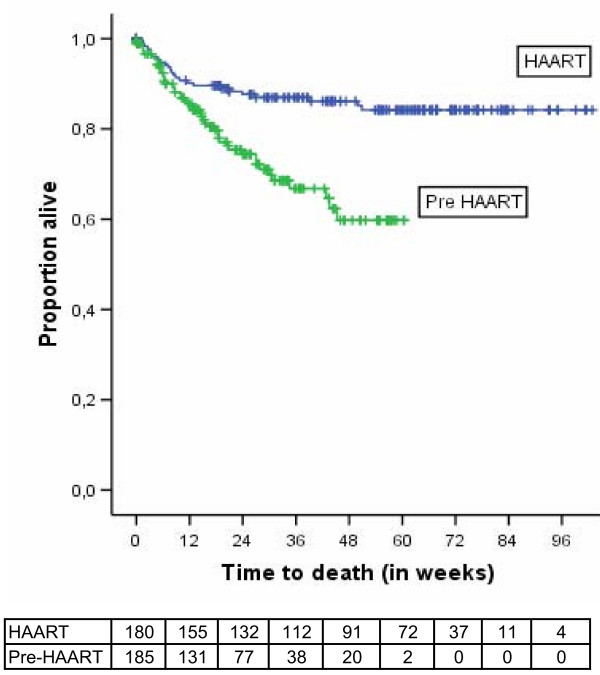
**Kaplan-Meier estimates of survival with and without HAART, Arba Minch Hospital, 2006**. This figure shows (i) the higher mortality rate in the untreated group, and (ii) the high early mortality in both groups. About 20 weeks after starting treatment, the mortality rate stabilized in the treated group, but in the untreated cohort there was steady increase in mortality. The longer curve of the HAART group shows the longer follow-up in the treated group.

**Figure 2:**
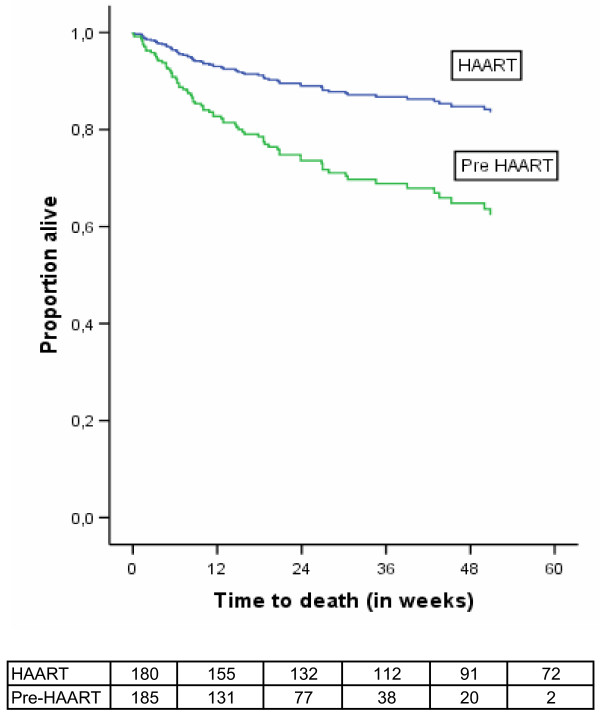
Survival curve according to adjusted Cox regression analysis shown in Table 3.

**Figure 3:**
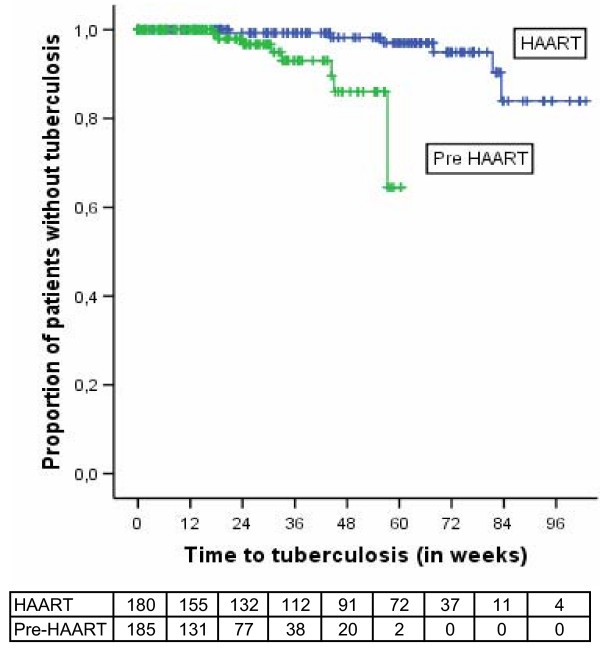
**Kaplan Meier estimates of tuberculosis-free survival in the two cohorts, Arba Minch Hospital, 2006**. This figure shows the high tuberculosis occurrence in the untreated group. Unlike the mortality rate which was highest in the early weeks, tuberculosis started at about 20 weeks after enrolment (pre-HAART) or after starting treatment (HAART). This could be because patients might have died even before being diagnosed. Additionally, it could be due to the time needed to diagnose new cases.

HAART resulted in a decrease of mortality (adjusted HR [95%CI] = 0.35 (0.19–0.63) P < 0.001). Figure 1 shows the Kaplan-Meier survival curves. Using Cox-regression analysis adjusting for oral thrush, diarrhea, total lymphocyte count, anaemia and low body mass index shows improved survival for patients receiving HAART (Figure 2). Tuberculosis incidence rate was also lower in the HAART group (adjusted HR [95%CI] = 0.11 [0.03–0.48]; P < 0.01). Figure 3 shows Kaplan Meier survival plots for tuberculosis occurrence.

Other factors predicting higher mortality rates among our patients were oral thrush, TLC <1200/ml, BMI less than 18.5 kg/m2, anaemia, WHO clinical stages III or IV and presence of prolonged diarrhea at baseline. However, when stratified by treatment group, only low TLC and low BMI were associated with increased mortality both in the pre-HAART (BMI, P = 0.016; TLC, P= 0.009) and in the HAART groups (BMI, P = 0.017; TLC, P = 0.039) while anaemia was not associated with increased mortality in either group (pre-HAART, log-rank = 2.2, P = 0.14; HAART, Log-rank = 2.1, P = 0.14). Tables 3 and 4 show the relative effect of selected variables on mortality and tuberculosis incidence rates.

**Table 3:** Hazard ratios (HR) of death according to Cox-regression analyses, Arba Minch Hospital, 2006

Factor	Crude HR (95%CI)	P-value	Adjusted HR (95%CI)	P-value
HAART (yes vs. no)	0.38 (0.23–0.62)	<0.001	0.35 (0.19–0.63)	<0.001
Oral thrush (yes vs. no)	2.64 (1.66–4.19)	<0.001	1.15 (0.90–2.63)	0.119
Diarrhea (yes vs. no)	1.84 (1.16–2.93)	0.010	1.43 (0.85–2.40)	0.172
TLC <1200 vs. > = 1200/ml	2.22 (1.30–3.79)	0.004	2.48 (1.32–4.66)	0.005
BMI (<18.5 vs. 18.5+kg/m^2^	2.50 (1.44–4.34)	0.001	1.83 (1.03–3.27)	0.040
Hgb (<10 vs. > = 10 g/dl)	2.26 (1.21–4.25)	0.010	1.15 (0.55–2.40)	0.710
WHO stage (III-IV vs. II)	3.57 (1.12–11.34)	0.030	2.74 (0.62–12.12)	0.184

BMI= body mass index; Hgb=haemoglobin; TLC=total lymphocyte count; WHO=World Health Organization

**Table 4:** Hazard ratios of tuberculosis incidence rate according to Cox-regression analysis, Arba Minch Hospital, 2006

Factor	Crude HR (95%CI)	P-value	Adjusted HR (95%CI)	P-value
HAART (Yes vs. No)	0.09 (0.02–0.37)	0.001	0.11 (0.03–0.48)	0.003
Fever (Yes vs. No)	5.66 (1.85–17.3)	0.002	5.28 (1.67–16.66)	0.005
Hgb (<10 g/dl vs. > = 10 g/dl)	9.24 (1.19–71.5)	0.033	5.77 (0.70–47.7)	0.104
Easy fatigability (Yes vs. No)	1.42 (0.47–4.26)	0.530	1.79 (0.58–5.51)	0.306

History of easy fatigability (Log-rank = 13.8; P < 0.001) and prolonged fever (Log-rank = 17.2; P < 0.001) predicted tuberculosis in the pre-HAART group but not in the HAART group (Log-rank = 0.06; P = 0.80 for fever and Log-rank = 2.3, P = 0.13 for easy fatigability). History of tuberculosis was not associated with increased tuberculosis incidence, neither in the pre-HAART group (Log-rank = 1.7; P = 0.19) nor in the HAART group (Log-rank = 2.1; P = 0.15).

## Discussion

We found that HAART decreased death and tuberculosis incidence rates in HIV infected Ethiopian patients. Mortality declined by 65% and tuberculosis incidence rate was reduced by almost 90%. Most of the deaths in the HAART cohort occurred within the first three months of therapy. Few patients experienced life-threatening drug side effects.

We did the study under routine care conditions in a typical Ethiopian district hospital. We were able to follow the patients and believe the results may be relevant to other resource-limited settings. The use of community agents in following up the patients was not only useful to secure complete data for the research, but proved to be helpful in the routine clinical work.

However, both the mortality and the tuberculosis incidence rates are higher in this cohort than in cohorts from the developed world and there is a need to improve the treatment routines. Particular attention should be on the early weeks of treatment when mortality is high. Unfortunately, many of our patients had advanced disease at time of diagnosis. Thus, to reduce mortality, HIV patients may need to be diagnosed earlier. Treatment programmes need to work closely with voluntary testing programmes, the tuberculosis control units and with the HIV prevention efforts.

Both patients and health workers should be aware that tuberculosis might occur among patients receiving HAART. Patients experiencing prolonged fever before treatment are at higher risk of being diagnosed with tuberculosis.

Our results are consistent with findings from the developed world when HAART was introduced. A protease inhibitor-containing HAART reduced mortality by 64% in an Italian cohort [8]. Similarly, a multicenter HAART trial reported a 62% decrease in mortality among patients with advanced HIV [9]. To our knowledge, no study has compared HAART with no treatment in an African setting. A study using virologic and immunological end points found that HAART was equally effective in African patients [5] and challenged an earlier report that virological failure was more common in Africans [10].

While improved survival is encouraging news, it is lower than in the developed world. A population based study from Ethiopia showed that adult mortality rate is 11.1 per 1000 PYO [11]. A background of high disease burden and advanced disease stage could explain the high mortality rate of 15.4 per 100 PYO among our patients. Since most of the deaths occurred within the first 12 weeks of treatment, factors associated with advanced disease or the immune reconstitution syndrome could be possible contributing causes [12]. Though we did not make any attempt to ascertain the specific causes death, we suspect undiagnosed tuberculosis either alone or as part of the immune constitution syndrome to be the cause for some of the early deaths. This could be particularly true for the majority of deaths that occurred at home. This highlights the need for improving the quality of patient management during the early phase of treatment, as has been shown in developed countries [2,13]. A more recent study confirmed the high early mortality in resource-poor settings and emphasized the need for timely diagnosis and treatment [14].

Doing studies in resource-limited settings as in Arba Minch is difficult and adds some limits on how the study was carried out. Because of limited funds, we were not able to do CD4 tests. Even if this essential test was not done, the treatment results were good and the incomplete laboratory set-up should not be used to deny HAART to patients in developing countries. The diagnosis of tuberculosis was based on clinical, radiological or sputum examination only. As we were not able to do tuberculosis cultures, we may have overestimated the number of tuberculosis cases diagnosed. As HAART is a lifelong treatment, we plan to follow the cohort and learn more about the long-term effects of the drugs and to learn more about drug adherence.

Recent evidence shows that HAART reduces the risk of tuberculosis by 70–90% [15-18], as shown in our study. However, despite the decrease in tuberculosis among patients treated with HAART, the rate is still high when compared to HIV-negative patients. In South Africa, the tuberculosis incidence rates in the HAART and in the pre-HAART cohort were 2.4 and 9.7 per 100 PYO, respectively [16] and compare well with our corresponding rates of 3.7 and 11.1 per 100 PYO. With a longer treatment period it is possible to decrease the tuberculosis incidence to about 1 per 100 PYO [19,20]. However, the tuberculosis incidence rates from Africa are still higher than 0.79 per 100 PYO reported from an Italian cohort [18].

These high tuberculosis incidence rates, when coupled with the improved survival, could lead to increased tuberculosis transmission in the community [18,21]. Thus, tuberculosis preventive strategies need to be strengthened with the rapid scale-up of HAART. Some of the suggested strategies include: early introduction of HAART, isoniazid (INH) prophylaxis and immune boosting mechanisms [15]. In the resource-limited settings such as in south Ethiopia, the INH prophylaxis seems the more possible alternative.

## Conclusion

HAART improved survival and decreased tuberculosis incidence to a level similar to that achieved in the developed countries during the early years of HAART. This relative reduction in mortality and tuberculosis is encouraging news. However, both the mortality and the tuberculosis incidence rates were higher in this resource-limited setting. Since most of the deaths occurred during the early weeks of treatment, attention should be paid to this part of the follow-up. The high tuberculosis incidence rate, when combined with the improved survival, may lead to increased tuberculosis transmission. This highlights the need for strengthening tuberculosis prevention efforts along with the scale-up of treatment programmes.

## Methods

### Study setting

Arba Minch Hospital, located 500 km south of Addis Ababa, is a general hospital with basic facilities for HIV care and treatment. The hospital has been doing HIV counselling and testing since the early 1990's and since January 2002, the services include basic tests to deliver antiretroviral drugs (ARVs) and to treat opportunistic infections [7]. In August 2003, Arba Minch hospital was among the first few public hospitals to start HAART in Ethiopia. The country did not have a policy on the use of ARVs until July 2002 [22].

As part of the preparation to start HAART in south Ethiopia, we registered and followed all consecutive HIV infected patients who visited the clinic since January 2003. During this pre-HAART period, patients were followed and treated for opportunistic infections. Since August 2003, we treated patients with HAART. Therefore, we had two cohorts of patients: the pre-HAART and the HAART cohorts.

### The Pre-HAART cohort

All adult treatment-naïve patients (age > = 15 years) with symptomatic HIV disease (WHO stage II to IV) who had follow-up in the HIV unit of the hospital between January 2003 and August 2003 were eligible for this analysis. At baseline, we examined patients and classified them according to the WHO staging [23]. A complete blood cell count (CBC) was done after the clinical staging. CD4 counts were not available. Chest X-ray and sputum for acid-fast bacilli were done when clinically indicated. Identified opportunistic diseases were treated according to the standard in the hospital. We repeated clinical examinations and CBCs every 12 weeks and encouraged the patients to visit the clinic whenever needed. The results of the pre-HAART follow-up, including patients with stage I disease, have been reported elsewhere [24].

### The HAART cohort

This study included all consecutive adult patients (age > = 15 years) treated with triple antiretroviral drug at Arba Minch hospital during the period August 2003 to August 2005. Recruitment into the treatment was based on the Ethiopian and the WHO treatment guidelines [7,25]. So, all HIV positive patients with WHO stage II-IV were eligible to be evaluated for treatment. In stage II, we started treatment if the patients had a total lymphocyte count (TLC) of less than 1200/mm^3^. The patient's willingness to be treated was another condition for inclusion.

At baseline, we recorded socio-demographic, clinical and laboratory data in a standardized patient record form. Eligible patients were counselled for drug adherence and their consent was obtained before drugs were prescribed.

### Approved antiretroviral drugs

Generic combinations of stavudine (d4T), lamivudine (3TC), nevirapine (NVP), zidovudine (ZDV) and efavirenz (EFV) are approved for use in district hospitals in Ethiopia [25]. The first-line drug combination of choice (d4T/3TC/NVP) was prescribed to all patients if there were no clear contraindications such as liver disease, intensive phase of antituberculosis treatment or allergy to any one of the components.

Stavudine was given as 40 mg or 30 mg (depending on body weight) twice daily; 3TC 150 mg twice daily; and NVP 200 mg daily for the first two weeks and then twice daily after that. ZDV was available only in combination with 3TC and it was given at a dose of 300 mg twice daily. The dose of EFV was 600 mg daily. EFV was mainly reserved for patients in the intensive phase of antituberculosis treatment. All drugs were given orally.

### Follow-up assessment

Following the baseline assessment, the patients were provided with more drugs on four-weekly basis. Each month, we assessed patients for drug side effects, treatment compliance, measured the body weights, and examined for new symptoms. Particular attention was given to diagnosis of tuberculosis. CBC, liver and renal tests were measured every 12 weeks.

### Follow-up at community level

Each month, a community agent visited the patients in their homes. These community agents had completed secondary school and had received extra training on HIV/AIDS. After each visit, they reported the status of each patient.

### End Points

The primary end point in this study was time to death. All non-accidental deaths were considered HIV-related. The secondary end point was time to diagnosis of tuberculosis. We defined and classified tuberculosis according to the national tuberculosis and leprosy control manual of Ethiopia [26]. Accordingly, we diagnosed smear positive pulmonary tuberculosis (PTB+) if two or more initial sputum examinations were positive for AFB, or one sputum positive for AFB plus radiographic abnormalities consistent with active TB as determined by a physician. Smear negative pulmonary tuberculosis (PTB-) was diagnosed if at least three sputum specimens negative for AFB, and radiologic abnormalities consistent with TB, and no response to a course of broad-spectrum antibiotics, and decision by a physician to treat with a full course of antituberculosis chemotherapy. EPTB refers to TB of organs other than the lungs, and diagnosis was based on strong clinical suspicion by a physician.

Patients were regarded as lost to follow-up if they did not attend the hospital within the previous 90 days and the community agent did not find the patient. We followed the patients until they died, moved out of the area or were lost to follow-up. We regarded the community agent to be the most reliable informant of outcome occurring at community level. The data clerk at the clinic reported hospital deaths.

For the HAART period, censoring time was defined as the earliest date of the following events: (i) if the person was alive and on treatment at the end of the study, August 9 2005; (ii) if the person was lost to follow up, the date of the last contact the patient had with the community agent; (iii) if the patient was transferred to another institution, the date of transfer; and (iv) if the patient stopped treatment, the last date of drug resupply plus two months.

Patients in the pre-HAART study were followed until they were lost, transferred, died, put on HAART, or reached last date of the pre-HAART follow-up (1 April 2004) whichever occurred first. We followed pre-HAART patients about 90 days into the HAART period until we got information about all the patients including those still on their 12-weekly appointment. We calculated time-to-event from the first date of clinic visit for the pre-HAART cohort and from the date HAART was started for the HAART cohort. Thus, the pre-HAART patients who joined the HAART group contributed person-time to both cohorts at different periods. All those started on HAART were considered treated irrespective of the outcome to approximate the intention-to-treat analysis of randomized trials.

### Statistical methods

To assess the event-free survival, we used the Kaplan-Meier method and the Log-rank test was used to test for the statistical significance. We also used the Cox-regression method to find out the effect of HAART on mortality and on tuberculosis incidence rates. We assessed the relative effect of the WHO clinical stages, oral thrush, diarrhoea, body mass index (BMI), anaemia and total lymphocyte count (TLC) in predicting mortality in both the pre-HAART and the HAART groups. Anaemia, history of prolonged fever, history of easy fatigability, history of tuberculosis, and the WHO clinical stage were also assessed as possible predictors of tuberculosis.

We used the cut-off value of 18.5 kg/m^2 ^for low BMI [27]. Since we do not have normal value for haemoglobin (Hgb) in the study area, we used an arbitrary cut off value of 10 g/dl to define anaemia. The cut off point for the TLC was 1200 cells/ml because of its clinical significance in starting HAART [25].

We calculated death rates as deaths per 100 person-years of observation (PYO) and the tuberculosis incidence rates as number of tuberculosis cases per 100 PYO. All patients were considered to be at risk of developing tuberculosis during follow-up.

We used SPSS for Windows version 13.0 (SPSS Inc, Chicago, USA.) for data analysis. All completed baseline and follow-up patient data were entered on the same day of examination or as soon as they were available. Statistical tests were considered significant if the two-sided P-value was <0.05.

### Ethical Considerations

The study protocol was approved by the Regional Committee for Medical Research Ethics in Norway and by the National Ethics Review Committee in Ethiopia. Patients gave informed consent for participating in the study.

## Competing interests

The author(s) declare that they have no competing interests.

## Authors' contributions

DJ, AN and BL designed the study. DJ recruited and followed the patients. DJ and BL analyzed the data. DJ, BL and AN drafted the manuscript and approved the final version.
